# The Association of Clinical Symptoms and Coexistent Clinical Conditions with Ophthalmic Manifesting in COVID-19 Patients

**DOI:** 10.22088/cjim.13.0.180

**Published:** 2022

**Authors:** Mansour Babaei, Masomeh Bayani, Maral Farzin, Seyed Ahmad Rasoulinejad

**Affiliations:** 1 Mobility Impairment Research Center, Health Research Institute, Babol University of Medical Sciences, Babol, Iran; 2 Clinical Research Development Unite of Rouhani Hospital, Babol University of Medical Sciences, Babol, Iran; 3 Department of Internal Medicine, School of Medicine, Babol University of Medical Sciences, Babol, Iran; 4 Infectious Diseases and Tropical Medicine Research Center, Health Research Institute, Babol University of Medical Sciences, Babol, Iran; 5 School of Medicine, Babol University of Medical Sciences, Babol, Iran; 6 Department of Ophthalmology, Ayatollah Rouhani Hospital, Babol University of Medical Sciences, Babol, Iran

**Keywords:** Ocular manifestations, COVID-19, Epiphora, Blurred vision, Eye redness, photophobia

## Abstract

**Background:**

The ocular symptoms are common manifestations in coronavirus infectious disease 2019 (COVID-19), which faces secondary complications and therapeutic challenges. Underlying diseases actuate the body to infectious diseases and their related manifestations through the aberration of metabolism and suppressing the immune system. This study aimed to investigate the correlation of underlying diseases and ocular manifestations in COVID-19 patients.

**Methods:**

This cross-sectional study was held on 108 hospitalized COVID-19 patients (confirmed by molecular detection) admitted to Rouhani hospital, Babol, Iran. Upon hospitalization, all clinical symptoms and underlying diseases were registered. Detailed clinical examinations regarding ophthalmological protocols were used to investigate the ocular symptoms. All analyses were performed by SPSS, version 25.

**Results:**

Our results showed that 26.67% of patients with at least one ocular symptom had hyperlipidemia, while 10.42% of patients without any ocular symptoms had hyperlipidemia (P=0.049). In this study, 97.81% of COVID-19 patients without epiphora had no thyroid disorders (hyper-/hypo-thyroidism), while 82.35% of COVID-19 patients with epiphora had not any thyroid disorders (P=0.012). Also, 75.00% of patients with blurred vision had diabetes mellitus, while 35.00% of patients without blurred vision suffered from diabetes mellitus. This difference was borderline significant (P=0.051). Other results showed that 13.04% of COVID-19 patients with eye redness suffer from myalgia, while 35.29% of patients without eye redness had myalgia (P=0.044). Also, 35.11% of COVID-19 patients without photophobia had myalgia, while none of the patients with photophobia had myalgia (P=0.005). Finally, 70.00% of patients with respiratory distress had at least one ocular symptom, while 43.10% of patients without respiratory distress had at least one ocular symptom (P=0.007).

**Conclusion:**

Some underlying diseases, e.g., hyperlipidemia, diabetes mellitus, and thyroid disorders, and some clinical symptoms in hospitalized patients, e.g., myalgia and respiratory distress, are correlated with ocular manifestations in COVID-19 patients.

According to the reports of the World Health Organization (WHO), coronavirus infectious disease 2019 (COVID-19) has infected more than 106 million people worldwide by February 2021, with a mortality of more than 2.31 million people (http://covid19.who.int). In the COVID-19 pandemic, there have been numerous reports of new complications in patients with the COVID-19. 

Gastrointestinal complications, joint and muscle pain, fever and cough, respiratory distress, and ocular complications are the most common manifestations in COVID-19 (1). The ocular manifesting is another complication in COVID-19 patients (2). The appearance of ocular symptoms in patients with COVID-19 is related to the presence of this virus in various tissues of the eye (3). The presence of the severe acute respiratory syndrome coronavirus 2 (SARS-CoV-2) in the conjunctiva has been confirmed using molecular detection techniques (4). Overall, different tissues of the eye are affected differently by infectious diseases associated with the coronavirus. Also, because of the connection between the eye mucosa and the upper respiratory tract through the nasolacrimal duct, the eye can be a way for respiratory viruses such as coronaviruses to enter and proliferate (5-7). Due to the pathogenic association of SARS-CoV-2 and ocular symptoms in patients with COVID-19, recognizing any possible association between COVID-19 patients' underlying diseases and ocular complications may accelerate the process of complete remission and prevent vision-related complications (8, 9). Therefore, ocular symptom management is critical in COVID-19 patients.

Underlying diseases play a critical role in the development of diseases and the presence of secondary complications. Some underlying diseases may cause some metabolism changes in the body. Also, some underlying diseases suppress the immune system, which makes the body prone to new infectious diseases. For example, diabetes, autoimmune diseases, and hyperlipidemia have a susceptive role in COVID-19. Also, various studies confirm the role of overweight and autoimmune diseases in the presence and severity of clinical symptoms in COVID-19 patients. Therefore, this study aimed to investigate the correlation of underlying diseases and ocular manifestations in COVID-19 patients. 

## Methods

**Patients and clinical examinations: **This cross-sectional study was conducted on 108 hospitalized COVID-19 patients (confirmed by molecular detection) admitted to Rouhani hospital, Babol, Iran, affiliated with Babol University of Medical Sciences (IR.MUBABOL.REC.1399.391). Upon hospitalization, all clinical symptoms were registered in terms of lethargy, loss of consciousness, fever, caught, dyspnea, headache, myalgia, vomiting, respiratory distress, diarrhea, decreased appetite, and weight loss. The medical history of patients was recorded in terms of hypertension, congestive heart failure (CHF), diabetes mellitus (DM), hyperlipidemia, ischemic heart disease (IHD), renal failure, coronary artery bypass grafting (CABG), angioplasty, thyroid disease (i.e., hyperthyroidism and hypothyroidism), and malignancy. Detailed clinical examinations regarding ophthalmological protocols investigated the ocular symptoms in terms of blurred vision, epiphora, discharge and exudate, redness, eye pain, photophobia, and itchy eyes. 

**Statistical analysis: **Fisher's Exact Test (FET) was used to investigate the correlation of clinical symptoms and underlying diseases with ocular symptoms in COVID-19 patients. All analyses were performed by SPSS version 25. The significant level was determined as 95% (P <0.05).

## Results

**Hyperlipidemia, diabetes, and thyroid disorders promote ocular symptoms in COVID-19 patients: **Our results show that hyperlipidemia is significantly more prevalent in COVID-19 patients with at least one ocular symptom compared to COVID-19 patients without any ocular manifestation (26.67% vs. 10.42%, p =0.049). As a result, 97.81% of COVID-19 patients without epiphora had not thyroid disorder (hyper-/hypo-thyroidism), while 82.35% of COVID-19 patients with epiphora had not any thyroid disorder (hyper-/hypo-thyroidism) (P =0.012). Also, 14.29% of COVID-19 patients with photophobia suffer from thyroid disorder (hyper-/hypo-thyroidism), while 3.19% of COVID-19 patients without photophobia had thyroid disorder (hyper-/hypo-thyroidism) (P=0.059). Our results showed that 75.00% of patients with blurred vision had diabetes mellitus, while 35.00% of patients without blurred vision suffered from diabetes mellitus. This difference was borderline significant (P=0.051) (table 1).

**Myalgia and respiratory distress are correlated with ocular manifestations: **Myalgia is more prevalent in patients with blurred vision (62.5%) than patients without blurred vision (28.00%), while this difference was borderline significant (P=0.055). Our results showed that 13.04% of COVID-19 patients with eye redness suffer from myalgia, while 35.29% of patients without eye redness had myalgia (P=0.044). Also, 35.11% of COVID-19 patients without photophobia had myalgia, while no one of patients with photophobia had myalgia (P=0.005). As a result, 29.41% of COVID-19 patients with epiphora suffer from respiratory distress, while 58.24% of COVID-19 patients without epiphora had respiratory distress (P=0.036). Also, 70.00% of patients with respiratory distress had at least one ocular symptom, while 43.10% of patients without respiratory distress had at least one ocular symptom (P=0.007) (table 2).

**Table 1:** Correlation of ocular symptoms and background diseases in COVID-19 patients

Underlying Disease	Status	Blurred Vision		Epiphora		Discharge and exudate		Redness		Eye pain		Photophobia		Itchy eyes		At least one ocular symptom	
		No	Yes	No	Yes	No	Yes	No	Yes	No	Yes	No	Yes	No	Yes	No	Yes
Hypertension	No (n =61)	57	4	52	9	56	5	51	10	57	4	55	6	56	5	31	30
	Yes (n =47)	43	4	39	8	43	4	34	13	44	3	39	8	43	4	17	30
	Total (n =108)	100	8	91	17	99	9	85	23	101	7	94	14	99	9	48	60
	P	0.726		0.794		1.000		0.165		1.000		0.387		1.000		0.172	
Congestive heart failure	No (n =107)	99	8	90	17	98	9	84	23	100	7	93	14	99	8	48	59
	Yes (n =1)	1	0	1	0	1	0	1	0	1	0	1	0	0	1	0	1
	Total (n =108)	100	8	91	17	99	9	85	23	101	7	94	14	99	9	48	60
	P	1.000		1.000		1.000		1.000		1.000		1.000		0.083		1.000	
Diabetes mellitus	No (n =67)	65	2	57	10	61	6	52	15	63	4	58	9	60	7	33	34
	Yes (n =41)	35	6	34	7	38	3	33	8	38	3	36	5	39	2	15	26
	Total (n =108)	100	8	91	17	99	9	85	23	101	7	94	14	99	9	48	60
	P	0.051		0.790		1.000		0.812		1.000		1.000		0.478		0.234	
Hyperlipidemia	No (n =87)	82	5	75	12	81	6	71	16	80	7	77	10	80	7	43	44
	Yes (n =21)	18	3	16	5	18	3	14	7	21	0	17	4	19	2	5	16
	Total (n =108)	100	8	91	17	99	9	85	23	101	7	94	14	99	9	48	60
	P	0.184		0.316		0.373		0.146		0.341		0.467		1.000		**0.049**	
Ischemic heart disease	No (n =83)	78	5	70	13	77	6	68	15	79	4	72	11	74	9	39	44
	Yes (n =25)	22	3	21	4	22	3	17	8	22	3	22	3	25	0	9	16
	Total (n =108)	100	8	91	17	99	9	85	23	101	7	94	14	99	9	48	60
	P	0.383		1.000		0.429		0.165		0.349		1.000		0.114		0.367	
Renal failure	No (n =102)	94	8	87	15	93	9	80	22	96	6	88	14	94	8	45	57
	Yes (n =5)	5	0	3	2	5	0	4	1	4	1	5	0	4	1	2	3
	Total (n =107)	99	8	90	17	98	9	84	23	100	7	93	14	98	9	47	60
	P	1.000		0.178		1.000		1.000		0.292		1.000		0.361		1.000	
Coronary Artery Bypass Grafting	No (n =98)	92	6	84	14	89	9	76	22	92	6	86	12	89	9	44	54
	Yes (n =9)	7	2	6	3	9	0	8	1	9	0	8	1	9	0	4	5
	Total (n =107)	99	8	90	17	98	9	84	23	101	6	94	13	98	9	48	59
	P	0.135		0.152		1.000		0.680		1.000		1.000		1.000		1.000	
Angioplasty	No (n =99)	92	7	82	17	91	8	77	22	93	6	86	13	91	8	44	55
	Yes (n =9)	8	1	9	0	8	1	8	1	8	1	8	1	8	1	4	5
	Total (n =108)	100	8	91	17	99	9	85	23	101	7	94	14	99	9	48	60
	P	0.514		0.349		0.557		0.681		0.466		1.000		0.557		1.000	
Thyroid disease	No (n =103)	95	8	89	14	94	9	80	23	96	7	91	12	95	8	47	56
	Hypothyroidism (n =4)	4	0	1	3	4	0	4	0	4	0	3	1	4	0	1	3
	Hyperthyroidism (n =1)	1	0	1	0	1	0	1	0	1	0	0	1	0	1	0	1
	Total (n =108)	100	8	91	17	99	9	85	23	101	7	94	14	99	9	48	60
	P	1.000		0.012		1.000		0.666		1.000		0.059		0.115		0.792	
Malignancy	No (n =100)	92	8	85	15	92	8	79	21	93	7	87	13	92	8	44	56
	Yes (n =7)	7	0	5	2	6	1	5	2	7	0	6	1	6	1	3	4
	Total (n =107)	99	8	90	17	98	9	84	23	100	7	93	14	98	9	47	60
	P	1.000		0.308		0.469		0.641		1.000		1.000		0.469		1.000	

**Table 2:** Correlation of ocular symptoms and clinical symptoms in COVID-19 patients

		Blurred Vision		Epiphora		Discharge and exudate		Redness		Eye pain		Photophobia		Itchy eyes		At least one ocular symptom	
		No	Yes	No	Yes	No	Yes	No	Yes	No	Yes	No	Yes	No	Yes	No	Yes
Lethargy	No (n =64)	61	3	55	9	61	3	51	13	58	6	57	7	57	7	30	34
	Yes (n =35)	30	5	29	6	31	4	27	8	34	1	29	6	33	2	14	21
	Total (n =99)	91	8	84	15	92	7	78	21	92	7	86	13	90	9	44	5
	P	0.127		0.772		0.240		0.800		0.416		0.535		0.486		0.534	
loss of consciousness	No (n =99)	91	8	84	15	92	7	79	20	94	5	85	14	91	8	45	54
	Yes (n =9)	9	0	7	2	7	2	6	3	7	2	9	0	8	1	3	6
	Total (n =108)	100	8	91	17	99	9	85	23	101	7	94	14	99	9	48	60
	P	1.000		0.631		0.164		0.398		0.104		0.602		0.557		0.728	
Fever	No (n =45)	41	4	37	8	39	6	34	11	43	2	40	5	42	3	19	26
	Yes (n =63)	59	4	54	9	60	3	51	12	58	5	54	9	57	6	29	34
	Total (n =108)	100	8	91	17	99	9	85	23	101	7	94	14	99	9	48	60
	P	0.717		0.789		0.160		0.634		0.697		0.774		0.732		0.844	
Caught	No (n =48)	45	3	41	7	43	5	38	10	45	3	43	5	43	5	23	25
	Yes (n =60)	55	5	50	10	56	4	47	13	56	4	51	9	56	4	25	35
	Total (n =108)	100	8	91	17	99	9	85	23	101	7	94	14	99	9	48	60
	P	0.730		0.797		0.507		1.000		1.000		0.572		0.507		0.562	
Dyspnea	No (n =103)	95	8	95	8	95	8	82	21	96	7	91	12	94	9	46	57
	Yes (n =5)	5	0	5	0	4	1	3	2	5	0	3	2	5	0	2	3
	Total (n =108)	100	8	100	8	99	9	85	23	101	7	94	14	99	9	48	60
	P	1.000		0.175		0.358		0.288		1.000		0.125		1.000		1.000	
Headache	No (n =92)	86	6	77	15	85	7	72	20	86	6	79	13	83	9	40	52
	Yes (n =16)	14	2	14	2	14	2	13	3	15	1	15	1	16	0	8	8
	Total (n =108)	100	8	91	17	99	9	85	23	101	7	94	14	99	9	48	60
	P	0.377		1.000		0.619		1.000		1.000		0.688		0.350		0.786	
Myalgia	No (n =75)	72	3	63	12	68	7	55	20	71	4	61	14	69	6	31	44
	Yes (n =33)	28	5	28	5	31	2	30	3	30	3	33	0	30	3	17	16
	Total (n =108)	100	8	91	17	99	9	85	23	101	7	94	14	99	9	48	60
	P	0.055		1.000		0.719		0.044		0.435		0.005		1.000		0.402	
Vomiting	No (n =96)	90	6	81	15	87	9	76	20	89	7	83	13	88	8	43	53
	Yes (n =12)	10	2	10	2	12	0	9	3	12	0	11	1	11	1	5	7
	Total (n =108)	100	8	91	17	99	9	85	23	101	7	94	14	99	9	48	60
	P	0.217		1.000		0.593		0.716		1.000		1.000		1.000		1.000	
Respiratory Distress	No (n =50)	45	5	38	12	46	4	32	18	47	3	43	7	44	6	15	35
	Yes (n =58)	55	3	53	5	53	5	53	5	54	4	51	7	55	3	33	25
	Total (n =108)	100	8	91	17	99	9	85	23	101	7	94	14	99	9	48	60
	P	0.467		0.036		1.000		0.001		1.0000		0.788		0.298		**0.007**	
Diarrhea	No (n =101)	94	7	85	16	93	8	79	22	95	6	88	13	92	9	45	56
	Yes (n =7)	6	1	6	1	6	1	6	1	6	1	6	1	7	0	3	4
	Total (n =108)	100	8	91	17	99	9	85	23	101	7	94	14	99	9	48	60
	P	0.426		1.000		0.466		1.000		0.383		1.000		1.000		1.000	
Decreased Appetite	No (n =87)	80	7	72	15	79	8	69	18	81	6	75	12	81	6	38	49
	Yes (n =21)	20	1	19	2	20	1	16	5	20	1	19	2	18	3	10	11
	Total (n =108)	100	8	91	17	99	9	85	23	101	7	94	14	99	9	48	60
	P	1.000		0.517		1.000		0.770		1.000		1.000		0.373		0.809	
Weight Loss	No (n =106)	98	8	89	17	98	8	84	22	99	7	93	13	97	9	48	58
	Yes (n =2)	2	0	2	0	1	1	1	1	2	0	1	1	2	0	0	2
	Total (n =108)	100	8	91	17	99	9	85	23	101	7	94	14	99	9	48	60
	P	1.000		1.000		1.000		0.382		1.000		0.244		1.000		0.502	

## Discussion

The presence of ocular symptoms in COVID-19 patients may be a sign of direct eye involvement with SARS-CoV-2 or secondary effects of cytokine-induced inflammation in these patients. In addition to creating new problems for the patient, the onset of ocular symptoms can complicate the treatment of ocular diseases in COVID-19 patients. Therefore, ocular symptom management in COVID-19 patients is essential. The underlying diseases suscept the body of COVID-19 patients to secondary manifestations, i.e., ocular symptoms, through affecting the metabolism and immune system responses. This study aimed to investigate the correlations of underlying diseases and ocular manifestations in COVID-19 patients to prevent the secondary complications of ocular diseases. 

Our results indicated that 19.44% of COVID-19 patients had hyperlipidemia. The study conducted by Palaiodimos et al. revealed that the prevalence of hyperlipidemia was 46.2% among COVID-19 patients (10). Since Hyperlipidemia induces inflammation, the presence of inflammation-related manifestations is expected in COVID-19 patients. Various studies established the role of inflammation and overresponse of the immune system in ocular manifestations (11-13). Our results demonstrated that 26.67% of patients with at least one ocular symptom had hyperlipidemia, while 10.42% of patients without any ocular symptoms had hyperlipidemia. Furthermore, Lee et al.’s study found no significant difference between the ocular symptom-positive group and the ocular symptom-negative group in terms of hyperlipidemia (P =1.00). Also, hyperlipidemia was not correlated with conjunctival congestion in COVID-19 patients (P =0.32) (14). There was no other study regarding the association of hyperlipidemia and ocular manifestations in COVID-19 patients. 

Our results showed that 75.00% of patients with blurred vision had diabetes mellitus, while 35.00% of patients without blurred vision have diabetes. Abrishami et al. found that 36% of COVID-19 patients without any ocular symptoms had diabetes mellitus, while 53.3% of patients with ocular symptoms had diabetes mellitus (15). Therefore, the ocular symptoms are more prevalent in diabetic COVID-19 patients compared to non-diabetics. Despite our study and Abrishami et al.'s study, the study of Lee et al. showed that there was no correlation between diabetes mellitus and ocular manifestations in COVID-19 patients (14). Hyper-/hypo-thyroidism is not more prevalent in COVID-19 patients. In other words, hyper-/hypo-thyroidism does not increase the chance of COVID-19 morbidity, but it induces ocular manifestation in infected patients. Our results showed that hyper-/hypo-thyroidism promotes COVID-19 patients to epiphora and photophobia. There was no study investigating thyroid disorders' impact as an underlying disease that affects immunity in ocular manifestations in COVID-19 patients. It should be noted that cytokine storm, as a hallmark of COVID-19, can affect the thyroid gland and promotes hypothyroidism (16). 

Our results demonstrated that myalgia is correlated with blurred vision in COVID-19 patients. As a finding, myalgia was more prevalent in patients with blurred vision (62.5%) than patients without blurred vision (28.00%). Despite the direct correlation between myalgia and blurred vision, there is a reverse correlation between myalgia with eye redness and photophobia. Our results showed that 13.04% of COVID-19 patients with eye redness had myalgia, while this number was 35.29% for patients without eye redness. Contrary to our results, in the study of Abrishami et al., there was no correlation between myalgia and ocular symptoms in COVID-19 patients (15). Our results did not find any correlation between dyspnea and ocular symptoms in COVID-19 patients. Our study is also in contrast with Abrishami et al. who found a significant correlation between dyspnea and ocular manifestation in COVID-19 patients (P=0.015) (15). In their experiment, 62% of patients with no ocular manifestations had dyspnea, while 40.2% of patients with ocular manifestations had dyspnea. Moreover, compared with our study, in the study of Abrishami et al., there was no correlation between cough, fever, headache, and respiratory distress with ocular manifestations in COVID-19 patients (15). Overall, our results established that some underlying diseases, e.g., hyperlipidemia, diabetes mellitus, and thyroid disorders, are correlated with ocular manifestation in COVID-19 patients. On the other hand, the presence of some clinical symptoms in hospitalized patients, e.g., myalgia and respiratory distress, are correlated with ocular manifestations in COVID-19 patients. Our findings can contribute to better management of ocular diseases in COVID-19 patients during this pandemic. The establishment of a correlation between underlying diseases and ocular manifestations in COVID-19 patients shows that the management of underlying metabolic diseases, e.g., hyperlipidemia, diabetes mellitus, and thyroid disorders, can prevent the ocular manifestations in COVID-19-infected cases. The management of mentioned metabolic diseases is more critical in COVID-19 patients with background ocular diseases. It is recommended that more COVID-19 patients be studied to investigate the correlation of ocular manifestations and underlying diseases on more patients to estimate the impact factor and odds ratio of underlying diseases on the presence of ocular manifestations in COVID-19 patients.
